# Snail2 promotes osteosarcoma cell motility through remodelling of the actin cytoskeleton and regulates tumor development

**DOI:** 10.1016/j.canlet.2013.01.027

**Published:** 2013-06-10

**Authors:** Amir-Shaya Sharili, Steve Allen, Ken Smith, Joanna Price, Imelda M. McGonnell

**Affiliations:** a Department of Comparative Biomedical Sciences, Royal Veterinary College, London, UK; b Department of Pathology and Pathogen Biology, Royal Veterinary College, London, UK; c Department of Clinical Veterinary Sciences, University of Bristol, Langford House, Langford, North Somerset, UK; d Barts and the London School of Medicine and Dentistry, Blizard Institute, 4 Newark Street, London E1 2AR, UK

**Keywords:** Osteosarcoma, Snail2, Migration, Invasion, Actin cytoskeleton, Tumorigenesis

## Abstract

The function of Snail2 in mesenchymal tumors is, to date unknown. Using knockdown and overexpression studies, we show that Snail2 regulates migration and invasion of osteosarcoma cells. Knockdown resulted in significantly decreased motility, remodelling of the actin cytoskeleton, and loss of cellular protrusions. Over-expression increased motility, formation of actin-rich cellular protrusions, and altered expression of some non-canonical Wnt pathway components whilst decreasing expression of the adhesion molecule OB-cadherin. Unexpectedly, knockdown also resulted in significantly smaller tumors in an *in vivo* CAM assay. Therefore Snail2 may be a potential therapeutic target for clinical intervention of osteosarcoma.

## Highlights

► Snail2 is over-expressed in osteosarcoma. ► Snail2 regulates osteosarcoma cell migration and invasion. ► Snail2 also regulates osteosarcoma tumor size. ► Snail2 may represent a therapeutic target for osteosarcoma.

## 1 Introduction

In epithelial tumor types (e.g. breast, lung and ovarian) the functions of the Snail zinc finger transcriptional repressors have been extensively studied [1]. In this context, the key function of Snail2 is similar to its function in embryonic epithelial tissues, namely the promotion of epithelial-to-mesenchymal transition (EMT) [2]. The mechanism of action is also similar, utilizing transcriptional repression of epithelial cellular adhesion molecules, including E-cadherin, thus allowing cells to break their cell to cell contacts [3–5], which is an early step in the process of EMT.

During embryonic development Snail2 is present in one tissue of mesenchymal origin, namely the developing long bone [6]. However its functions in this tissue are largely unknown. Interestingly expression is lost with age and in post-natal bone *in vivo*, Snail2 is absent (unpublished observations).

In a recent study, we demonstrated that Snail2 is expressed in long bone canine osteosarcomas; tumors of mesenchymal origin [7]. Furthermore our study also showed that there was a strong correlation between levels of Snail2 and grade (malignancy) of these osteosarcomas. This suggests that the re-expression of high levels of Snail2 in this tumor type may, in part, be responsible for increasing malignancy. Since osteosarcomas are mesenchymal tumors the function of Snail2 cannot be to drive changes in epithelial cell adhesion during EMT, suggesting that it most likely has other unknown functions in these, and possibly other, mesenchymally derived tumor types.

In order to investigate the function of Snail2 in osteosarcoma, we generated stable cell lines in which loss of Snail2 function was achieved using small interfering RNA and gain of function using CMV promoter driven over-expression. The motility of these tumor cells in vitro was assessed using a scratch assay and tumor forming ability together with vascular invasion determined in an *in vivo* model. Knockdown of Snail2 resulted in reduced motility while over expression of Snail2 resulted in increased motility. These changes in motility were associated with changes in the polymerization of the actin cytoskeleton and in focal adhesions as well as altered expression of Wnt5a, sFRP2 and osteoblast cadherin (OB-Cad). Reduction of Snail2 expression also resulted in reduced tumor forming ability in an *in vivo* assay. These data indicate a role for Snail2 in both motility and tumor formation.

## 2 Materials and methods

### 2.1 Establishment of stable osteosarcoma cell lines

Stable Snail2 cell lines were derived from canine D-17 and human Saos-2 osteosarcoma cell lines. Knock-down cell lines were produced by stable integration of shRNA producing plasmid vector, pLVX-shRNA2 (Clontech), using a previously characterized human Snail2 sequence (5′-GGACCACAGTGGCTCAGAA-3′) [8], also present in dog. Control vector contained a target sequence for eGFP. For Snail2 overexpression, the coding sequence of human Snail2, minus the stop codon, was inserted into pcDNA3.1 (Invitrogen) in frame with eGFP, producing a Snail2-eGFP fusion protein. Control vector contained the eGFP coding sequence. Cells were transfected with construct, plated in 100 mm culture dishes and selected with G418 and presence of GFP. Clonal colonies (2–3) of positive cells were ring cloned and individually amplified. A representative clone from each cell line was included. Overexpression and down-regulation of Snail2 was confirmed by immunofluorescent and qRT-PCR analysis. Cells from passages 4–10 were used in subsequent experiments.

### 2.2 Cell culture

Cell lines were grown in Dulbecco’s modified Eagle’s medium supplemented with 10% (v/v) fetal calf serum, 2 mM l-glutamine, 100 units/ml penicillin and 100 mg/ml streptomycin. For scratch assays and qRT-PCR, 2.5 × 10^5^ cells were plated onto 12-well plates and 60 mm culture dishes respectively. For immunofluorescence 5 × 10^4^ cells were plated onto 13 mm diameter thermanox coverslips (Nunc, Rochester, NY). For Wnt5a blocking experiments *in vitro*, Saos-2 cells overexpressing Snail2 were plated onto 13 mm diameter coverslips and treated for either 4 or 24 h with anti-Wnt5a antibody (2 μg/ml; R&D Systems, [9]. Cells were fixed in 4% paraformaldehyde and their actin cytoskeleton visualized using Rhodamine pahalloidin (Invitrogen Molecular Probes, UK)

### 2.3 Scratch assays

Confluent cell monolayers were wounded with a pipette tip to obtain two perpendicular wounds, forming a cross shape. Wounds were photographed at 0, 24 and 72 h using an inverted microscope (Leica, Solms, Germany). Average distances between wound edges were calculated by measuring the uncovered wound area and dividing by the width of the field of view. Distance migrated was calculated by subtracting the average distance between wound edges from that at time 0. For each experiment a total of 12 wounds were measured per group, and each experiment was repeated three times.

### 2.4 *In vivo* cell invasion assays

Fertilized white leghorn chicken eggs (Henry Stewart, UK) were incubated at 37 °C. On day 9 of development [10] the chorio-allantoic membrane (CAM) surface was gently lacerated with filter paper, and a plastic ring (6 mm inner diameter) placed on this region. 25 μl of medium containing 3 × 10^5^ control or Snail2 knockdown cells was added to the ring and the eggs re-incubated for a further 7 days before CAMs were excised and fixed in 4% PFA. Tumor size and cell motility were assessed using a Nikon SMZ1500 microscope and DS-2Mv digital fluorescent camera (Nikon Instruments Inc., Japan). Tumor areas from two separate experiments were measured using ImageJ software (NIH, Maryland, US).

### 2.5 Immunofluorescence

Cells were fixed with 4% PFA for 10 min, and incubated on ice for 10 min in permeabilization buffer (20 mM Hepes, 300 mM Sucrose, 50 mM NaCl, 0.5% TX-100, 3 mM MgCl_2_, 0.05% Sodium Azide, pH 7.0). Cells were blocked in 10% Calf Serum and incubated with primary antibody overnight at 4 °C. Primary antibodies used were rabbit anti-Snail2 (4 mg/ml; Santa Cruz, Inc., USA.) and rabbit anti-Paxillin (4 μg/ml; Santa Cruz). Coverslips were washed and incubated for 45 min with biotin conjugated goat anti-rabbit secondary antibody (Dako, Glostrup, Denmark) followed by Strepavidin, Alexa Fluor® 555 or 633 (Invitrogen Molecular Probes, UK). Rhodamine–phalloidin staining was used to visualize actin (Invitrogen Molecular Probes, UK). Imaging was performed with a Zeiss LSM 510 laser scanning confocal microscope (Carl Zeiss, Inc., Thornwood, NY) or Leica DM4000B light microscope (Leica, Solms, Germany).

### 2.6 Quantitative real-time RT-PCR

Total RNA was isolated from cells using an RNeasy Plus Mini Kit (Qiagen). cDNA was synthesized using Superscript II Reverse transcriptase (Invitrogen Ltd., Paisley, Scotland, UK) and random hexamer primers. Quantitative real time RT-PCR (qRT-PCR) was carried out as previously described [11] using QuantiTect SYBR Green PCR kit and Opticon 2 LightCycler (MJ Research, Waltham, MA). Primers used were against Snail2, OB-Cadherin, Wnt5A, sFRP2 and the housekeeping genes β-actin, GAPDH and 18S (sequences and conditions in supplemental information). A relative standard curve was constructed for Snail2, OB-cadherin, Wnt5A, sFRP2 and the housekeeping genes using serial dilutions of their amplicons, and these standard curves were included in each run. Standards were run in duplicate and samples in triplicate. The expression levels for all the genes analyzed were normalized to β-actin, GAPDH and 18S.

### 2.7 Statistical analysis

Data are presented as mean ± standard deviation. Statistical comparison of each Snail2 modified cell line and their appropriate control cell line was performed using the Student’s *t* test in Microsoft Excel. In all cases, *P* < 0.05 was considered significant.

## 3 Results

### 3.1 Generation of stable Snail2 over-expressing/knock-down osteosarcoma cell lines

To investigate the functional role of Snail2 in osteosarcoma tumorigenesis, stable cell lines were produced which either overexpressed or had reduced levels of Snail2. Overexpression and knock-down were confirmed by immunohistochemistry and qRT-PCR. Antibody labeling showed that levels of nuclear Snail2 protein were increased in both D-17 and Saos-2 overexpressing cells compared to controls (Fig. 1a). In contrast, knockdown cells showed reduced levels of nuclear Snail2 expression compared to controls in both cell lines (Fig. 1a). Analysis of Snail2 expression by qRT-PCR in D-17 cell lines matched the results seen for immunostaining (Fig. 1b and c). Snail2 transcript levels were increased and decreased for overexpression and knockdown lines respectively, and these changes were maintained over time in culture (Fig. 1b and c). In Saos-2 Snail2 overexpressing cells, increase in levels of Snail2 transcripts were only evident at later passage numbers (Fig. 1d), even though both low and high passage cells produced exogenous GFP tagged Snail2 from the inserted vector. Equally while immunostaining showed a decrease in Snail2 protein levels in shRNA cells (a decrease similar to that seen in another study using the same target sequence in an ovarian cell line [8]), qRT-PCR analysis showed no decrease (Fig. 1e). This may be due to an imperfect match between target and sequence which can produce inhibition without mRNA cleavage. Cell lines are known to possess SNPs and other genetic changes that may affect perfect matching and cleavage [12–14] and thus result in the apparent discrepancy between the mRNA and protein levels.

### 3.2 Snail2 modifies osteosarcoma cell morphology

Control cell lines maintained the same osteoblast-like phenotype as parental Saos-2 (human Fig. 2a: A and B and E and F) and D-17 (canine Fig. 2b: A and B and E and F) osteosarcoma cell lines. However, D-17 Snail2 shRNA cells showed a loss of normal osteoblast morphology, losing their characteristic spindle shape and becoming more polygonal or stellate (compare Fig. 2b: E and G), while Saos-2 Snail2 shRNA cells appeared relatively unchanged (compare Fig. 2a: E and G). Cell morphology was largely normal in D-17 cells overexpressing Snail2 (compare Fig. 2b: A and C), however Saos-2 cells overexpressing Snail2 showed an abnormal amoeboid appearance (compare Fig. 2a: A and C).

### 3.3 Snail2 regulates osteosarcoma cell motility

Scratch assays were performed to investigate changes in cell motility in Snail2 overexpressing and siRNA osteosarcoma cell lines. Knock-down of Snail2 in both canine and human osteosarcoma cells resulted in a significant reduction in motility from the wound edge compared to control cells (Fig. 3a and b). Overexpression of Snail2 significantly increased motility in Saos-2 but not D-17 osteosarcoma cells (Fig. 3a and b). Actin staining of Snail2 modified cells at the wound edge revealed morphological modifications. Control cells at the leading edge showed prominent cytoplasmic protrusions into which actin stress fibers extended to the tip, in the direction of migration (Fig. 3c; panels E and G (Saos2), M and O (D-17). Saos2 Snail2 overexpressing cells had a markedly different morphology and actin distribution. Cells at the leading edge had increased numbers of smaller protrusions containing condensed actin at the tip. In general, the actin cytoskeleton appeared disorganized in these cells with few, if any, stress fibers (Fig. 3c; panel F). In comparison, Saos-2 Snail2 knockdown cells at the leading edge had less well defined protrusions compared to controls and more prominent and intensely labeled stress fibers (Fig. 3c; panel H).

D-17 Snail2 overexpressing cells had similar morphology to controls, however there appeared to be less prominent stress fibers (Fig. 3c; panel N). In D-17 Snail2 knockdown cells, the vast majority did not form any protrusions. In those that did, actin fibers did not fully extend into these structures and were not always arranged in the direction of migration. (Fig. 3c; panel P).

### 3.4 Altered gene expression following Snail2 modulation

qRT-PCR analysis showed that Saos-2 cells overexpressing Snail2 had markedly reduced levels of OB-Cad expression (Fig. 4a), while decreasing levels of Snail2 increased OB-Cad expression. Furthermore Wnt5a showed an increase (Fig. 4c) while its antagonist sFRP2 decreased in Snail2 overexpressing osteosarcoma cells compared to controls (Fig. 4b). Expression of these two genes was not affected by knockdown of Snail2 (Fig. 4b and c).

Thus stable overexpression of Snail2 in Saos-2 cells leads to repression of OB-cadherin, which likely results in weaker cell–cell adhesion combined with an increase in pro-migratory non-canonical Wnt signaling due to increased Wnt5a expression which is compounded by decreased expression of its antagonist sFRP2.

### 3.5 Snail2 changes osteosarcoma cell focal adhesions and the cytoskeleton

In D-17 cells, downregulation of Snail2 was associated with a disorganized cytoskeletal architecture (Fig. 3c; panel L), however the number and size of focal adhesions appeared normal (Fig. 5B). Conversely in D-17 cells with increased Snail2 the actin cytoskeleton appeared normally organized (Fig. 3c; panel J), but had fewer stress fibers. In contrast, more focal adhesions were evident (Fig. 5D)

In Saos2 cells, down-regulation of Snail2 had little effect on cytoskeletal architecture (Fig. 3c; panel H) but resulted in greater numbers of larger focal adhesions (Fig. 5F). Conversely cells with increased levels of Snail2 had fewer actin cables but had a cortical distribution of actin as well as condensed actin appearing at points of cell–cell contact (Fig. 3c; panel F), similar to the distribution of actin seen in amoeboid cell migration [15]. Furthermore, paxillin was not detectable by immunostaining (Fig. 5H) indicating lack of focal adhesions, which may explain the observed weak adherence to substrate in this cell line. This suggests that one function of Snail2 in osteosarcoma cells is to regulate and/or organize cell adhesion and the actin cytoskeleton. In order to determine whether Snail2 regulates the actin cytoskeleton directly through Wnt5a, we treated Snail2 overexpressing cells with a Wnt5a blocking antibody [9]. Visualization of the actin cytoskeleton showed that neither short (4 h) nor long (24 h) treatment with this antibody had any observable effect on the actin cytoskeleton (Supplemental Fig. 1). This would suggest that cytoskeletal rearrangement is not a direct response to upregulation of Wnt5a.

### 3.6 Knock-down of Snail2 inhibits tumor development and cell invasiveness

The chick chorio-allantoic membrane (CAM) assay was used to study tumor formation and invasiveness in an *in vivo* model. Control cells formed prominent tumors and were able to invade the stroma, enter and migrate along the vasculature of the CAM (Fig. 6a; A, B, C and H, I, J). In contrast, D-17 and Saos-2 Snail2 knockdown cells were largely restricted to the region of implantation with little invasion of surrounding stroma and vasculature (Fig. 6a; D–F and K–M). Control Saos-2 cells developed a primary tumor at the site of implantation in all cases (2.57 mm^2^ ± 1.37; *n* = 12 Fig. 6a; A, B, and G), whereas the majority of Saos-2 Snail2 knock-down cells did not form a tumor (*n* = 7/12), and those that did formed markedly smaller and less dense tumors (1.01 mm^2^ ± 0.74; *P* < 0.001) (Fig. 6a; D, E, and G). Similarly, control D-17 cells developed a primary tumor at the site of implantation in all cases (3.77 mm^2^ ± 2.97; *n* = 9 Fig. 6a; I, J, and N). Almost 40% of canine knockdown cells failed to form a tumor and those that did (*n* = 7/11) formed markedly smaller and less dense tumors (1.28 mm^2^ ± 1.27; *P* < 0.05) (Fig. 6a; L, M, and N).

Histological examination of CAM graft tumors derived from control D-17 and Saos-2 cells revealed a population of mesenchymal osteosarcoma cells separated by chondroid/osteoid matrix, indicative of chondroblastic osteosarcoma (Fig. 6b; A and C). Conversely, tumors from D-17 and Saos-2 knock-down cells revealed a population of early mesenchymal cells contiguous with each other separated by minimal matrix (Fig. 6b; B and D).

## 4 Discussion

In humans and canines approximately 80% of osteosarcomas originate in the appendicular skeleton [16,17] and these are usually more aggressive than those originating at other skeletal sites [18]. Long bone osteosarcomas cause local skeletal and soft tissue destruction and are highly metastatic [19] with 20% of human patients showing clinically detectable metastases on initial presentation [20]. Despite advances in clinical management of osteosarcoma, the prognosis for osteosarcoma patients remains poor, with a reported 5 year survival rate of approximately 60% in metastatic disease [21], even when using chemotherapy as an adjuvant therapy [22].

To date there are no reliable biomarkers that predict clinical outcome or could be used in diagnosis to tailor treatments to individual patients. Therefore in order to improve diagnosis and treatment a better understanding of the biology of this disease is critical. In a recent study we demonstrated a correlation between Snail2 expression and tumor grade [7], which suggests that Snail2 may be involved in osteosarcoma progression, and therefore identifies it as a possible target for therapeutic intervention.

Snail2 is an established mediator of malignancy in epithelial tumors where it induces epithelial–mesenchymal transition (EMT), promoting both onset and progression of the disease [1]. However in osteosarcoma there is no requirement for EMT, as these tumors are mesenchymal in origin [23], so the function of Snail2 in these tumors remains unknown. We therefore generated osteosarcoma cell lines, with both increased and decreased levels of Snail2 protein, to investigate this.

We assessed the ability of these cells to migrate both *in vitro* and *in vivo*. In the osteosarcoma cell lines tested, decreasing the levels of Snail2 significantly decreased cell motility, demonstrating that Snail2 is required for migration. Conversely, increasing levels of Snail2 promoted motility in human Saos-2 cells but not in D-17 canine cells, demonstrating intrinsic differences between these cell lines. Saos-2 cells are derived from a primary osteosarcoma and are not highly metastatic. In contrast, D-17 cells are derived from a metastatic osteosarcoma of the lung and therefore may already be migrating at their maximum rate, which cannot be enhanced by additional Snail2 activity. Alternatively Snail2 activity could be saturated in these cells. In addition to migration, the CAM assay assessed the ability of Snail2 knockdown cells to invade stroma and intravasate, key steps in cancer cell metastasis. Control Saos-2 and D-17 cells were able to invade and intravasate while Snail2 knockdown attenuated these abilities. While the exact mechanism underlying these changes is not clear, it may be related to the control of MMP expression as Snail2 mediates the upregulation of MMP2 and MMP9 in a range of other cancers [24–26].

To further investigate the molecular mechanisms driving changes in motility, we examined members of two key pathways linked to Snail expression in both development and cancer. Snail2 is well known to inhibit expression of adhesion molecules such as E-cadherin, increasing cell migration due to reduced cell–cell adhesion [27]. E-cadherin is not highly expressed in osteosarcoma cells [28], however Snail2 may also regulate expression of other cadherins, such as mesenchymal cadherin (OB-cadherin/CDH11) [29], known to be expressed in osteosarcomas [29]. We have shown that expression of OB-cad is indeed inhibited by Snail2, which would promote cell migration and that knockdown of Snail2 allows for upregulation of OB-cad. In keeping with this, it has previously been shown that overexpression of OB-cadherin in osteosarcomas inhibits migration and reduces metastasis [30]. Furthermore a higher level of expression of OB-cadherin/CDH11 has been correlated with increased patient survival in osteosarcoma [31].

The second molecule investigated, Wnt5a, has been described as a tumor suppressor [32]. However, more recent evidence suggests it is a potent inducer of cell motility in a number of tumor types and cell lines, including osteosarcoma [33]. A recent paper has implied a link between another member of the Snail2 family (Snail1) and Wnt5a expression in Saos-2 cells [34]. Overexpression of Snail2 increases Wnt5a expression in Saos-2 cells and it is likely that this has a role in the increased motility observed. Previous analysis of the role of Wnt5a in gastric cancer cell lines indicated that loss of Wnt5a resulted in changes in the actin cytoskeleton [35]. In order to confirm whether Snail2 modulates cytoskeletal structure directly through Wnt5a, Saos-2 overexpressing cells were treated with a Wnt5a blocking antibody [9]. This resulted in no observable changes in cytoskeletal arrangement, suggesting that the effect of Snail2 on cytoskeletal reorganization is not a direct response to Wnt5a. It is therefore possible that Wnt5a is not the driving force for cytoskeletal changes and that the effect of Snail2 on the cytoskeleton is mediated by other mechanisms such as Rho-GTPases. In this context, Snail1 induced motility has been very recently reported to be mediated by Rho GTPases [36]. This raises the potential that Snail2 may also promote motility via Rho GTPases in osteosarcoma cells.

The lack of effect seen with Wnt5a neutralizing antibody does not rule out a role for Snail2 in maintaining and/organizing the cytoskeleton in link with cell motility. Therefore we examined in detail the cytoskeleton and focal adhesions.

In Saos-2 cells with decreased levels of Snail2, there were more prominent actin cables however they had fewer cellular protrusions, indicating a less migratory phenotype despite having more focal adhesions. In contrast, cells overexpressing Snail2, had regions of condensed actin in numerous small protrusions, reminiscent of structures described as invadopodia in metastatic cells [37] and indeed these cells were highly motile. Strikingly, they lacked paxillin containing focal adhesions.

Similar to Saos-2 cells, D-17 cell lines had fewer cellular protrusions when Snail2 levels were decreased, but no change in focal adhesions. Unlike the human cells, there was little effect on either the actin cytoskeleton or focal adhesions when Snail2 levels were increased and these cells did not have increased motility. This suggests that Snail2 has a prominent role in directing actin polymerization to form cell protrusions. The formation of cellular protrusions is largely controlled by actin related protein complexes (Arp 2/3) and actin severing proteins at the leading edge [38]. While Snail2 has been linked to disorganization of the actin cytoskeleton in pancreatic cancer cells [26], its role in cellular protrusions (i.e. lamellopidia, filopodia) has not been explored and this warrants further investigation.

Changes in focal adhesions will also alter cell migration dynamics. However the changes in focal adhesion in the human cells would initially appear to be contradictory to their migratory phenotype. It has previously been shown that knockdown of paxillin in highly metastatic osteosarcoma sub-lines M112 and 132 inhibits migration [39], which is in direct contrast to our observation that loss of paxillin in Saos-2 cells correlates with increased motility. However fibroblasts derived from paxillin knockout mice retained the ability to migrate [38], suggesting that paxillin expression is not a direct correlate with migration. However, loss of paxillin/focal adhesions may explain changes in morphology of Snail2 overexpressing cells, as paxillin deficient cells have previously been reported to have similar disorganized cortical cytoskeleton and delayed spreading in culture [40].

Another finding pertinent to the non-canonical Wnt signaling pathway is that overexpression of Snail2 reduces the expression of sFRP2, an inhibitor of Wnt5a signaling [41]. sFRP2 has not previously been identified as a target for Snail2 transcriptional repression in either physiological or pathological settings. The net result of downregulating this gene would be potentiation of the Wnt5a signal. Indeed, in cervical cancer it has been shown that expression of sFRP2 attenuates Wnt signaling and suppresses cancer cell growth [42]. Knockdown of Snail2 did not affect either sFRP2 or Wnt5a expression. This may suggest that these genes are not direct targets of Snail2.

A decrease in Snail2 expression also resulted in the generation of smaller tumors in the CAM assay. Reducing Snail2 expression reduced osteosarcoma cell migration and increased OB-cad expression, thus increasing cell–cell adhesion, which should promote tumor formation. However, cell–cell adhesion is not the only factor that drives tumorigenesis and the tumor microenvironment and matrix scaffold composition (such as collagen and fibronectin) is paramount for tumor formation [21,43]. Thus Snail2 may also regulate the expression of these proteins in osteosarcomas. Further studies are required to determine if this theory is correct.

Collectively, this study shows for the first time the requirement for Snail2 for motility and tissue invasion in human and canine osteosarcoma cells. Furthermore we also show that decreasing the levels of Snail2 impaired tumor development *in vivo*. Thus, the clinical benefits of selectively blocking Snail2 in patients with osteosarcoma may be twofold, as it may decrease metastasis, which is a leading cause of death, and also inhibit tumor growth.

## Figures and Tables

**Supplementary Figure 1:**
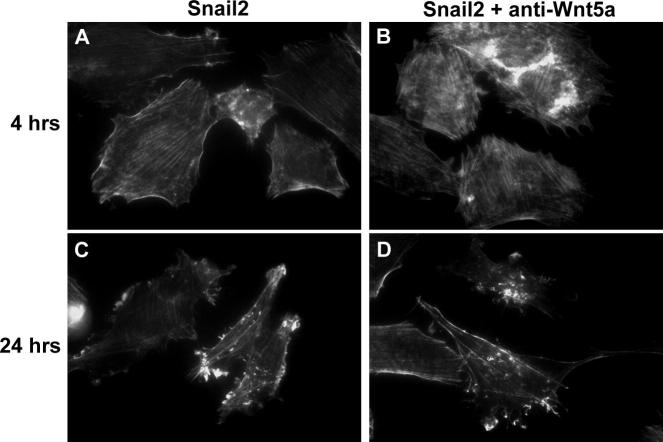


**Fig. 1:**
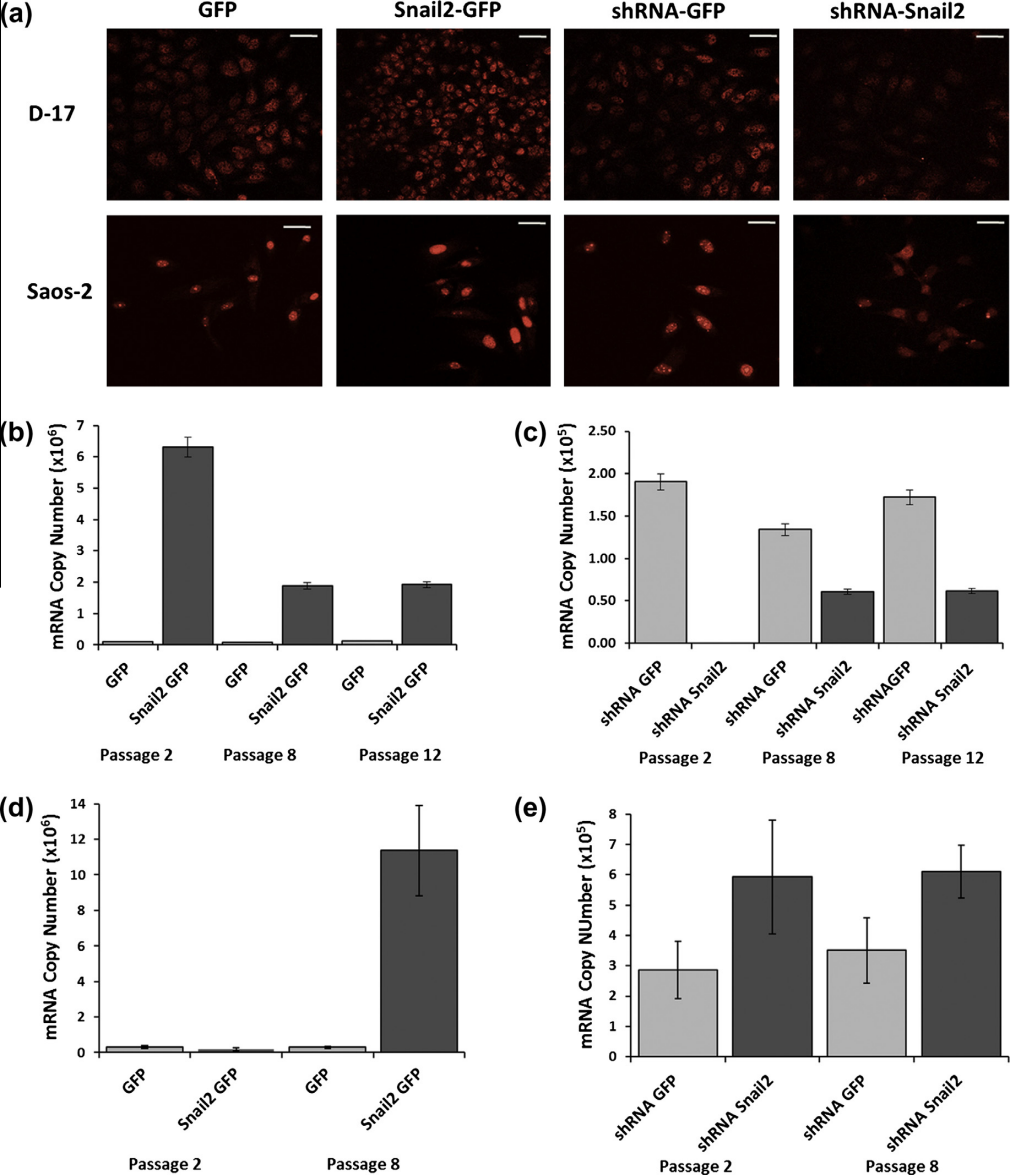
Immunofluorescent and qRT-PCR analysis of Snail2 expression levels in transgenic cell lines. (a) immunofluorescent localization of Snail2 protein in human and canine control (GFP) and Snail2 overexpressing (Snail2-GFP), control (shRNA-GFP) and Snail2 knockdown (shRNA-Snail2) osteosarcoma cells. Nuclear Snail2 levels were increased in Snail2-GFP and decreased in shRNA-Snail2 cells compared with controls GFP and shRNA-GFP expressing cells. Scale bars = 50 μm. (b–e) Total RNA was extracted and Snail2 mRNA expression levels measured by qRT-PCR. The expression levels were normalized to housekeeping genes, and results are expressed as mRNA copy numbers. In D17 cells Snail2 mRNA transcript levels were increased in Snail2-GFP (b) and decreased in shRNA-Snail2 (c) cells compared with controls (GFP and shRNA-GFP). These were stable over 12 passages. In Saos-2 cells, transcript levels for Snail2 were increased by passage 8 (d) but were not decreased with shRNA for Snail2 at either passage analyzed (e). Results are shown as mean ± SD of three replicates.

**Fig. 2:**
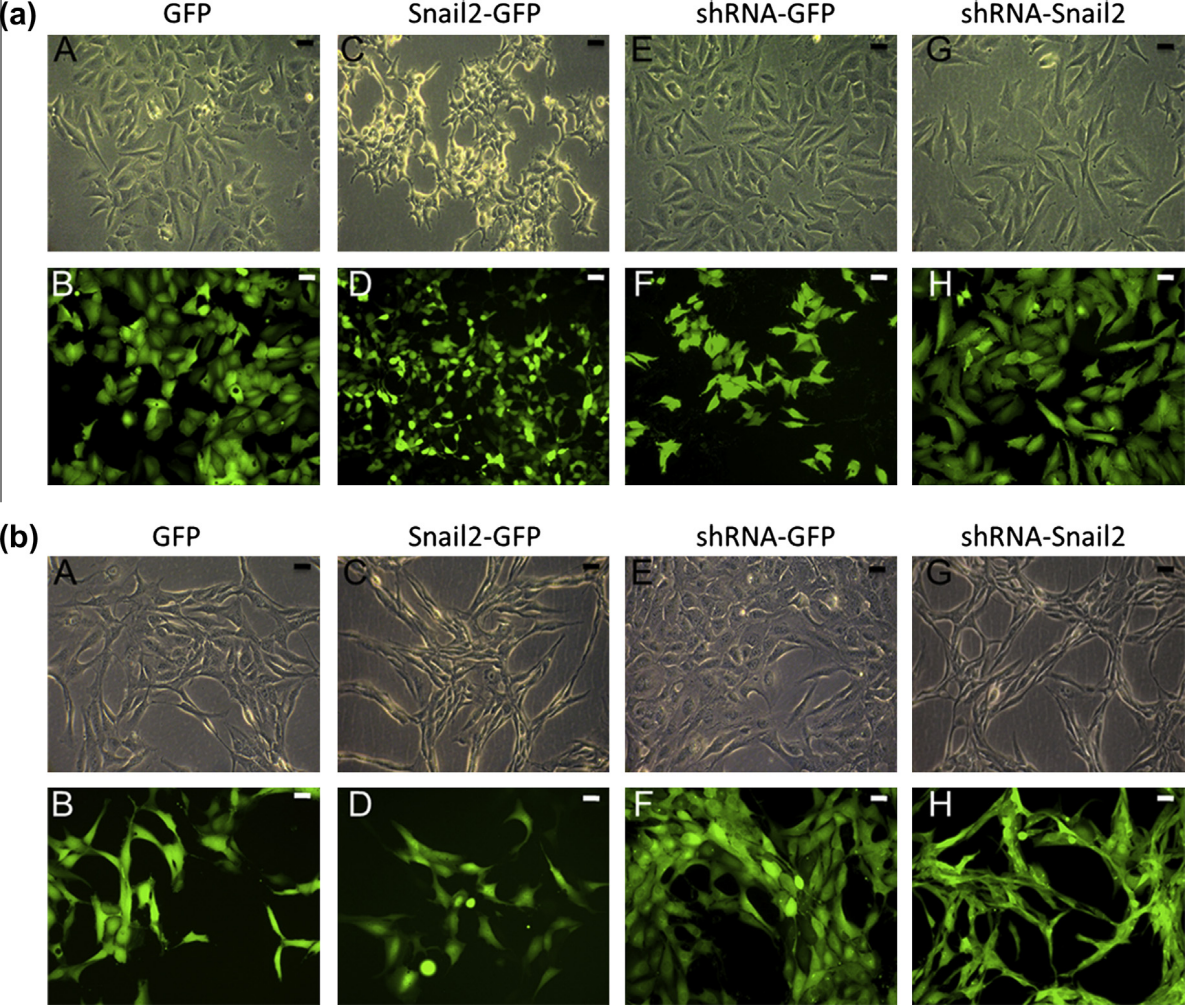
Snail2 overexpressing/knock-down human osteosarcoma cells show changes in morphology. Phase and fluorescent images of human Saos-2 (a) and canine D-17 (b) transgenic osteosarcoma cell lines expressing GFP. (a) Human Saos-2 Snail2 overexpressing cells (C and D) appeared smaller and more polygonal when compared to control cells (A and B). Saos2 knockdown (shRNA-Snail2) cells (G and H) showed no detectable changes in morphology compared to controls (E and F). (b) Canine D-17 overexpressing (Snail2-GFP) (C and D) and control (GFP) cells (A and B) showed a spindle shaped morphology, characteristic of osteoblastic cells. Canine Snail2 knockdown cells (shRNA-Snail2) (G and H) however acquired an elongated morphology compared to controls (E and F).

**Fig. 3:**
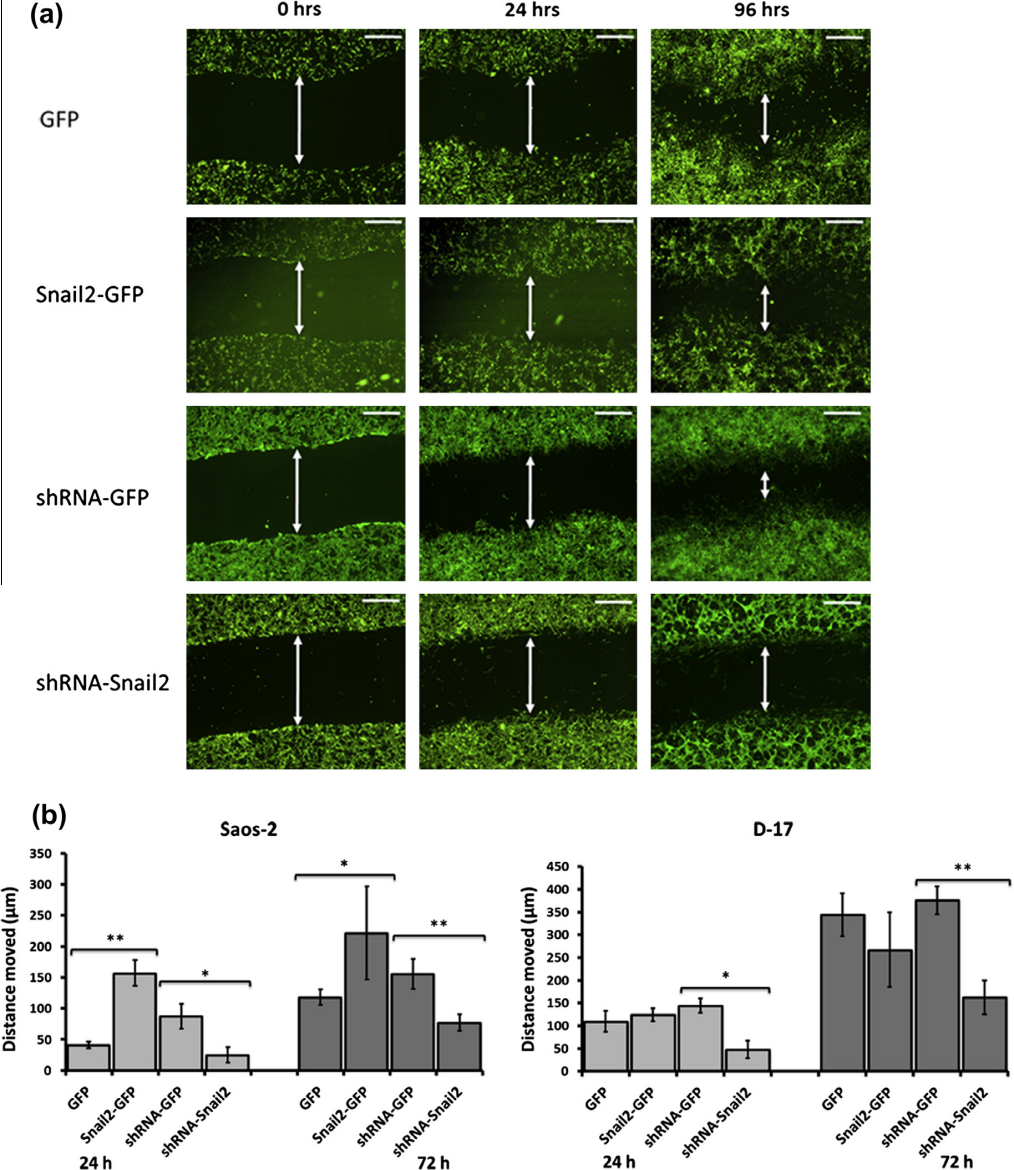
Snail2 knockdown human and canine osteosarcoma cells show decreased motility. (a) representative images of scratch assays in Saos-2 cells; control, (GFP) and (shRNA-GFP), and Snail2 overexpressing (Slug-GFP) and knock-down (shRNA-Snail2) cells at 0, 24 and 72 h. Double headed arrows indicate wound edges. Snail2 down-regulating cells showed a decreased motility compared with control. Over-expressing cells were more motile. (b) Graphical representation of average distance moved in Snail2 over-expressing/knock-down human and canine cells and controls with time. Results show mean ± SD of 12 wounds. ^*^P < 0.05 and ^**^P < 0.001. Scale bars = 50 μm. (c) Rhodamine–Phalloidin stained cultures of wounded human (A–H) and canine (I–P) Snail2 expressing, knockdown and control osteosarcoma cells. Human (Saos-2) Snail2 overexpressing cells showed a modified morphology with condensed regions of actin cytoskeleton at the tips of many cells (B and F) compared to their controls (A and E), however they still migrated as a coherent group. Human Snail2 knockdown cells (D and H) showed similar cell morphology and actin cytoskeleton to that seen in controls (C and G). In canine (D17) Snail2 overexpressing cells (J and N) the cell morphology and actin cytoskeleton was comparable to that seen in the controls (I and M). Canine Snail2 knockdown cells (L and P) did not migrate as a coherent group but as small groups of cells which lacked a leading edge and directionality when compared with controls (K and O). Scale bars = 10 μm.

**Fig. 3c:**
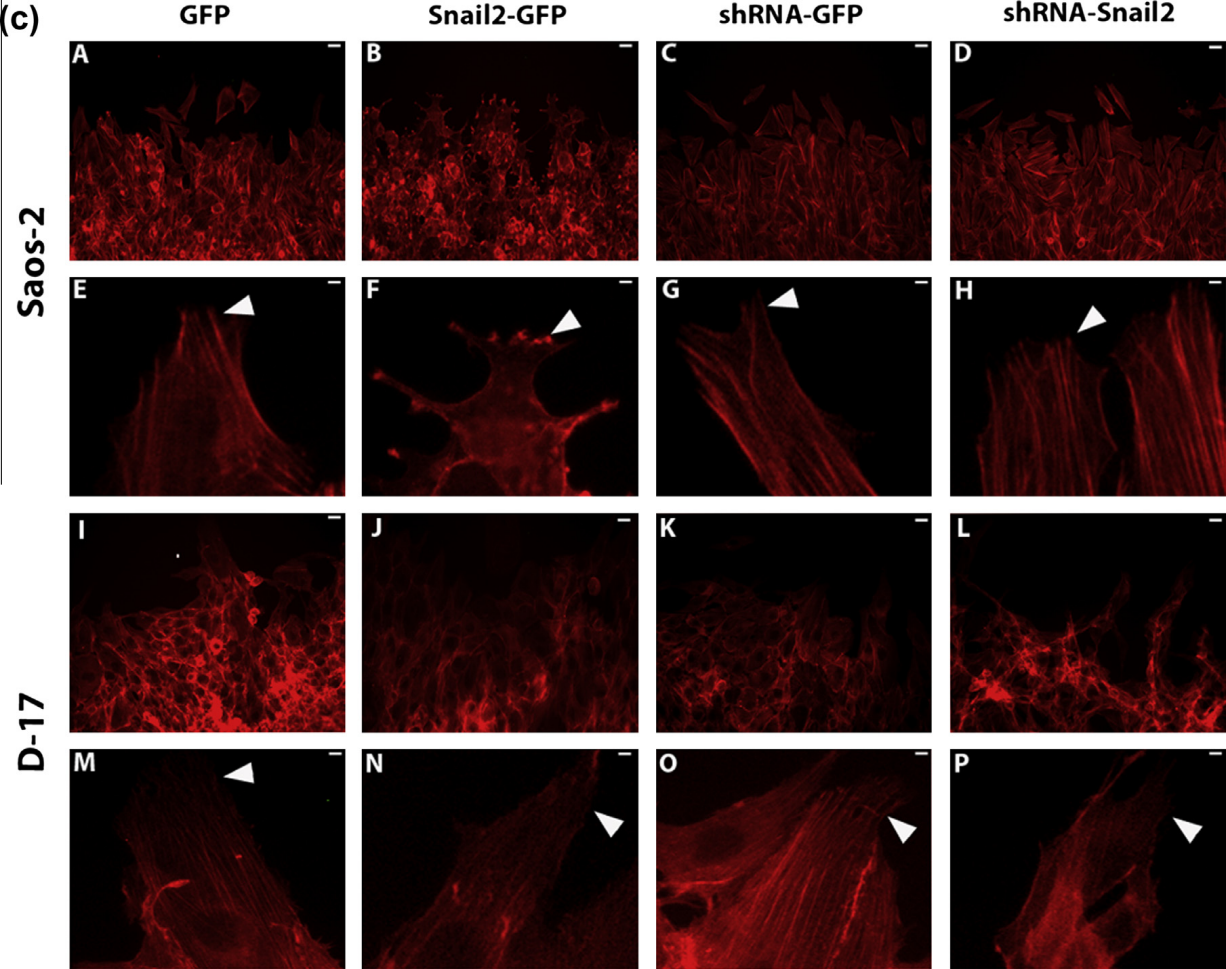


**Fig. 4:**
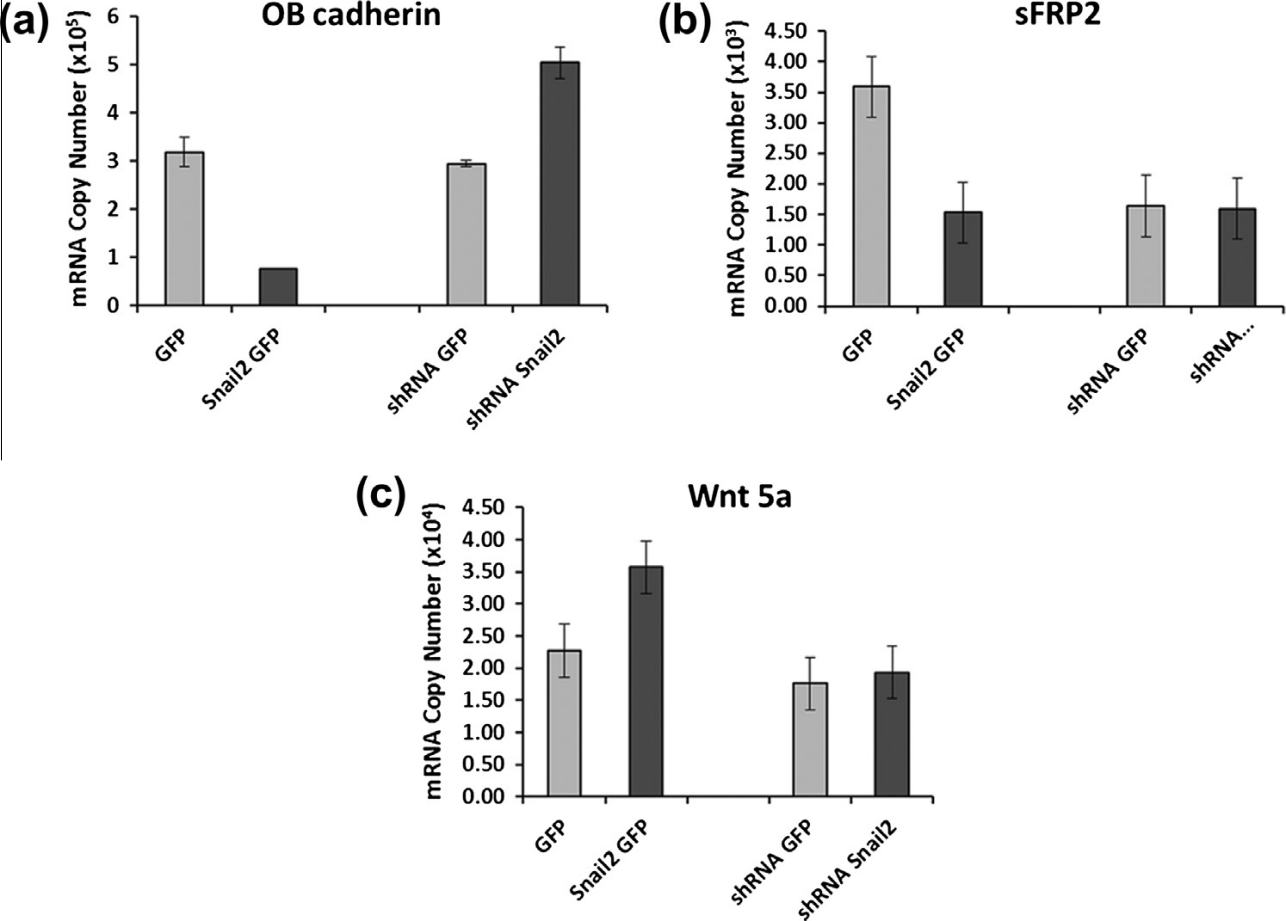
mRNA expression of OB-cad, sFRP2 and Wnt5a in Snail2 overexpressing/down regulating human osteosarcoma cells. Real time PCR revealed that OB-cadherin (a) and sFRP2 (b) transcript levels decreased and Wnt5a (c) increased following Snail2 over-expression (Snail2-GFP) compared with controls (GFP). Snail2 knockdown resulted in increased OB-cadherin but no alteration in sFRP2 or Wnt5a transcript levels.

**Fig. 5:**
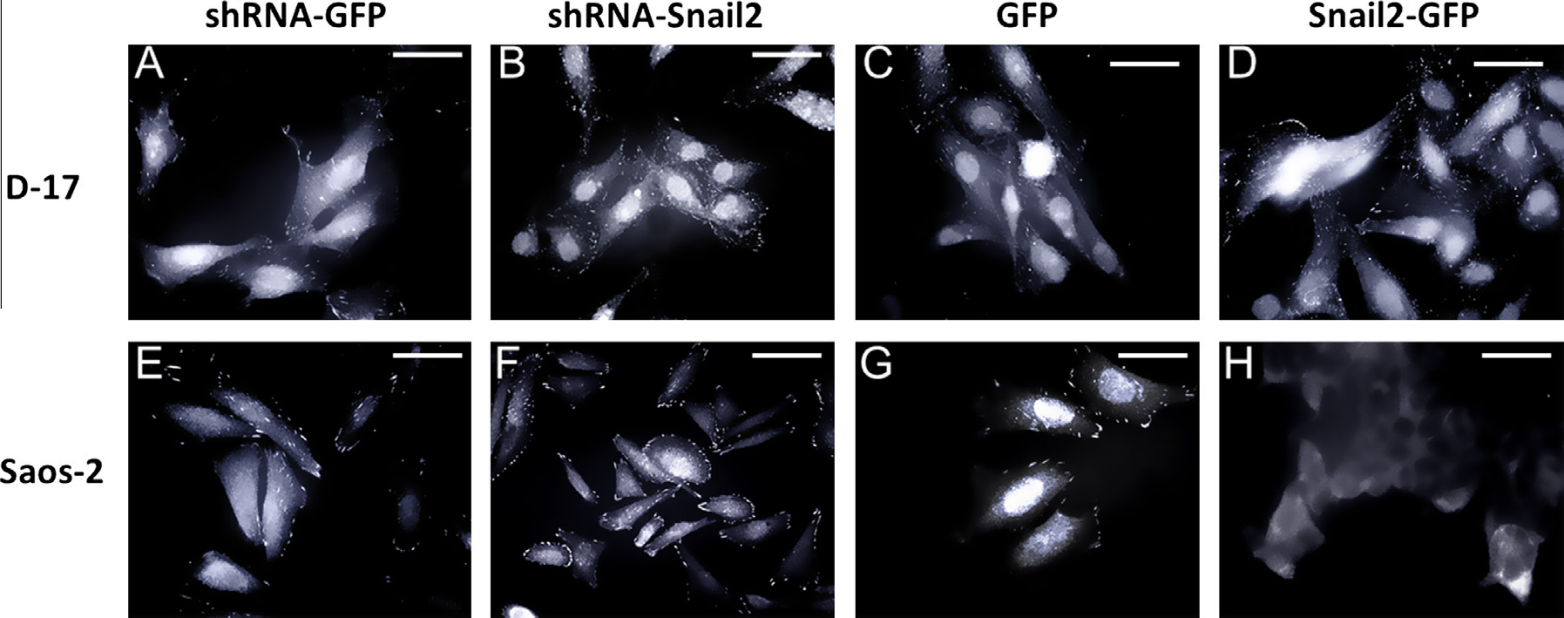
Effect of Snail2 overexpression/knock-down on focal adhesions. Fluorescent images of paxillin containing focal adhesions (A–H). Snail2 knockdown canine osteosarcoma cells showed no marked differences in focal adhesions (B) compared to their controls (A). Snail2 human knockdown cells showed more marked focal adhesions (F) than controls (E). Overexpressing canine cells showed an increased number of focal adhesions (D) compared to control (C). Human overexpressing cells showed no detectable focal adhesions (H), compared to their controls (G). Scale bars = 50 μm.

**Fig. 6:**
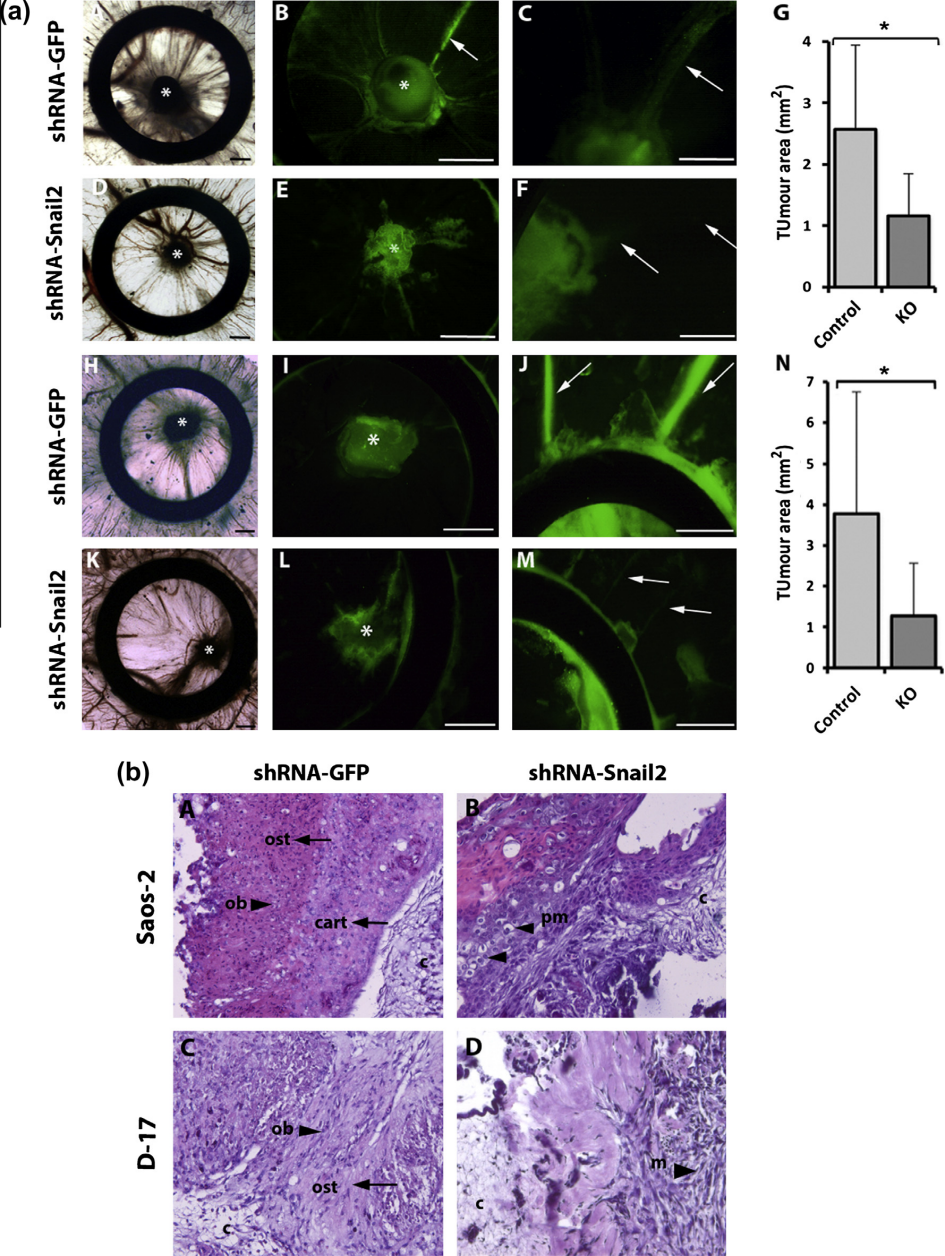
Knock-down of Snail2 decreases osteosarcoma growth and cell invasiveness *in vivo*. (a) Representative images of tumors from control (shRNA-GFP) and knock-down (shRNA-Snail2) canine and human cell lines grown on the CAM. A, D, H, and K: bright field images showing representative examples of rings containing tumors derived from Snail2 control and knock-down osteosarcoma cells (outlined) (10× magnification). B, E, I, and L: higher magnification fluorescent images of A, D, H and K (30× magnification) respectively. C, F, J, and M: fluorescent images showing GFP expressing cells invading blood vessels in control but not Snail2 knock-down tumors (arrows). Visualization of CAM vessels (arrows) showing GFP-expressing control cells inside the vasculature outside the ring (J). Note that Knock-down cells have not intravasted the CAM vessels (arrows) (F and M). G and N: graphs illustrating the mean tumor area. ^*^P < 0.05. Scale bars = 1 mm. (b) A: Saos-2 control tumor. Chondroblastic osteosarcoma with pale bluish chondroid (cart; arrow) and eosinophilic pink osteoid (ost; arrow) matrix. B: Saos-2 Snail2 knockdown tumor. Large polygonal cells with minimal matrix produced (arrowheads). C: D-17 control tumor. Spindle shaped osteoblast like cells (arrow head) separated by bony matrix. D: D-17 Snail2 knockdown tumor. Population of early mesenchymal cells contiguous with each other and not separated by matrix. ob: osteoblast cells; Ost: osteoid; cart: cartilage; pm: polygonal mesenchymal cells; m: mesenchymal cells; c: CAM.
