# Supplementary material for: Snail2 promotes osteosarcoma cell motility through remodelling of the actin cytoskeleton and regulates tumor development
Source: Cancer Lett. 2013 Jun 10;333(2):170–9. doi: 10.1016/j.canlet.2013.01.027 (PMC3644682; doi:10.1016/j.canlet.2013.01.027)
Supplement: Supplementary data 1 [file mmc1.docx]

**Supplemental**

Human/Dog Snail2 (sense 5’- AGAGCATTTGCAGACAGGTC-3’, antisense 5’- AGCCAGATTCCTCATGTTTG-3’)

human OB-Cadherin (sense 5’- ccagttcttcgtgatagagg-3’, antisense 5’- ATGTTCCCATCACCAGAGTC-3’),

Wnt 5A (sense 5’-GGCTGGAAGTGCAATGTCTTC-3’, antisense 5’ -TGTGCAGGTTCATGAGGATG-3’),

sFRP2 (sense 5’- GAGGAAGCTCCAAAGGTATG-3’, antisense 5’ -ATGCGCTTGAACTCTCTCTG-3’),

β-actin (sense 5’- CATGTTCGAGACCTTCAACACC-3’, antisense 5’ -CGTACCCCTCGTAGATGGGC-3’) .

PCR conditions were as follows: 1 cycle, 15 min, 95°C; 30 cycles, 30s, 94°C; 45s, 62°C; 30s, 72°C; 1 cycle, 10 min, 72°C.
